# Ganoderma lucidum Prevents Cisplatin-Induced Nephrotoxicity through Inhibition of Epidermal Growth Factor Receptor Signaling and Autophagy-Mediated Apoptosis

**DOI:** 10.1155/2020/4932587

**Published:** 2020-07-06

**Authors:** Yasmen F. Mahran, Hanan M. Hassan

**Affiliations:** ^1^Department of Pharmaceutical Sciences, Faculty of Pharmacy, Princess Nourah bint Abdulrahman University, Riyadh, Saudi Arabia; ^2^Department of Pharmacology & Toxicology, Faculty of Pharmacy, Ain Shams University, Cairo, Egypt; ^3^Department of Biochemistry, Faculty of Pharmacy, Delta University for Science and Technology, International Coastal Road, Gamasa City, Egypt

## Abstract

**Background:**

Cisplatin (cis-diaminedichloroplatinum, CDDP) is a broad-spectrum antineoplastic agent. However, CDDP has been blamed for its nephrotoxicity, which is the main dose-limiting adverse effect. Ganoderma lucidum (GL), a medicinal mushroom, has antioxidant and inflammatory activities. Therefore, this study is aimed at finding out the potential nephroprotection of GL against CDDP-induced nephrotoxicity in rats and the possible molecular mechanisms including the EGFR downstream signaling, apoptosis, and autophagy.

**Methods:**

Rats were given GL (500 mg/kg) for 10 days and a single injection of CDDP (12 mg/kg, i.p).

**Results:**

Nephrotoxicity was evidenced by a significant increase in renal indices and oxidative stress markers. Additionally, CDDP showed a plethora of inflammatory and apoptotic responses as evidenced by a profound increase of HMGB-1, NF-*κ*B, and caspase-3 expressions, whereas administration of GL significantly improved all these indices as well as the histopathological insults. Renal expression of EGFR showed a similar trend after GL administration. Furthermore, activation of autophagy protein, LC3 II, was found to be involved in GL-mediated nephroprotection correlated with the downregulation of apoptotic signaling, caspase-3 and terminal deoxynucleotidyl transferase (TDT) renal expressions.

**Conclusion:**

These results suggest that GL might have improved CDDP-induced nephrotoxicity through antioxidant, anti-inflammatory, and autophagy-mediated apoptosis mechanisms and that inhibition of EGFR signaling might be involved in nephroprotection.

## 1. Introduction

Cisplatin (cis-diaminedichloroplatinum, CDDP) is a frontline broad-spectrum antineoplastic agent [1]. It is widely used in clinical practice against various solid tumors such as head and neck cancer [2], ovarian cancer [3], bladder cancer [4], and breast cancer [5]. The anticancer action of CDDP is predominantly mediated via the formation of DNA adducts that activate various signaling mechanisms including cell cycle arrest and apoptosis [6]. However, albeit its effectiveness, CDDP has been blamed for its nephrotoxicity, the main dose-limiting adverse effect, seen in approximately one-third of the patients [7, 8]. Nephrotoxicity is elaborated by ample manifestations such as increased serum creatinine as well as blood urea nitrogen (BUN) levels and decreased renal blood flow, which reflects tubular dysfunction [9, 10]. Moreover, several mechanisms are involved in nephrotoxicity induced by CDDP such as the formation of toxic DNA adducts, lipid peroxidation, and depletion of reduced glutathione (GSH) [11, 12]. These events cause necrosis and mitochondrial vacuolization in renal proximal tubular cells, which are known to accumulate a high amount of CDDP [6]. It was reported that necrotic tubular cells release high-mobility group box-1 (HMGB-1), which induces further inflammatory response through activation of nuclear factor kappa B (NF-*κ*B) [13] leading to acute kidney injury (AKI) [14, 15]. Therefore, suppressing the oxidative and inflammatory-mediated tubular cell death is considered a good promising approach to prevent and treat CDDP-induced nephrotoxicity. Although tremendous approaches have been investigated for alleviating CDDP-induced nephrotoxicity, none of these approaches did completely and successfully protect the kidneys [13, 16]. Therefore, it is still necessary to identify novel effective strategies to minimize the renal cytotoxicity of CDDP. Of all these approaches used, natural compounds have been shown to provide promising protection [17–19].

Ganoderma lucidum (GL), a functional medicine and food, is one of the most highly regarded traditional Chinese medicines due to its ability to strengthen human health [20]. Modern pharmacological and clinical investigations have demonstrated that GL has multifarious pharmacological properties, such as antibacterial [21], antioxidant [22], and anti-inflammatory effects in different *in vivo* models [23, 24] in addition to its hepatoprotective and antitumor properties [25]. Indeed, GL possesses a wide variety of bioactive molecules like terpenoids, phenolic compounds, and polysaccharides, which have attracted attention in recent years due to their extensive antioxidation and anti-inflammation [22, 26, 27]. Some studies have shown that GL could protect the kidney against different models of acute nephropathy such as ischemia-reperfusion injury [28], diabetic nephropathy [29], and adriamycin-induced nephropathy [30]. Moreover, one study reported that GL terpenes protected against CDDP-induced nephrotoxicity through antioxidative effect; however, the mechanisms underlying this protection were not fully elucidated and still unclear [31]. Therefore, elaborating the mechanisms by which GL may protect CDDP renal injury is highly needed.

Indeed, ample pieces of evidence had explored the involvement of multiple cell death and survival pathways in CDDP nephrotoxicity [32]. However, the mechanisms involved in these pathways are controversial. Some studies reported the prosurvival role of autophagy in nephrotoxic models of CDDP [33], while Wada and his coworkers [34] suggested that erlotinib could ameliorate CDDP nephrotoxicity via inhibition of the epidermal growth factor (EGFR)/AKT downstream signaling pathway, which has a potential regulatory effect on autophagy [35]. Moreover, it is promising to find that ganoderic acid could target the EGFR downstream signaling transduction [36, 37]. Further elucidation of different interrelated signaling pathways involved in the CDDP-induced nephrotoxicity amendment is highly warranted. Consequently, this present study was conducted to find answers to the following questions. (1) Does GL produce significant nephroprotection against CDDP-induced nephrotoxicity in rats? (2) If so, what are the possible molecular mechanisms underlying this nephroprotective effect? (3) Is the autophagy/apoptosis interrelation involved in GL promising protection? (4) Does EGFR signaling contribute in GL nephroprotection?

## 2. Materials and Methods

### 2.1. Materials

Ganoderma lucidum was purchased from DXN Pharmaceutical SDN (BHD, Malaysia). CDDP was obtained from (Sigma Chemical Co., St. Louis, MO, USA). All other chemicals and solvents were of the highest grade commercially available.

### 2.2. Assessment of Total Phenolic Content, Total Flavonoid Content, and Total Antioxidant Capacities of GL Powder

Total phenolic content (TPC) was determined according to Folin–Ciocalteu reagent method reported by Lin et al. [38]. First, 0.1 ml aliquots of the GL in distilled water (0.1 g/ml) were mixed with 2.8 ml of distilled water, 2 ml of 2% (*w*/*v*) sodium carbonate, and 0.1 ml of 50% (*v*/*v*) of Folin–Ciocalteu reagent. Then, the obtained mixture was incubated at room temperature for half an hour and the absorbance was measured against distilled water at 750 nm, using a standard curve of gallic acid (0-200 mg/l). TPC value was expressed as milligram gallic acid equivalent (GAE)/g based on the dry weight. Results showed 133 mg GAE/g of GL powder.

Total flavonoid content (TFC) of GL powder was determined using aluminum chloride method which was described by Chang and coworkers [39], and the value was expressed as milligram quercetin equivalent (QE)/g based on the dry weight. Results showed 24 mg QE/g of GL powder.

Total antioxidant capacity of GL powder was evaluated using 2,20-azino-bis (3-ethyl benzothiazoline-6-sulfonic acid) (ABTS) assay method reported by Lissi et al. ABTS free radical-scavenging capacity of GL solution (1 mg/ml) was measured and expressed as percentage inhibition using ascorbic acid (20 *μ*l, 2 mM) solution as a standard antioxidant (positive control). GL showed a percentage inhibition of 58% against 88% of ascorbic acids [40].

### 2.3. Animals

The study was conducted according to ethical guidelines of the Ethical Committee of the Faculty of Pharmacy, University of Delta for Sciences and Technology Gamasa City, Egypt, approval number FPDU1/2020. The Guidelines for the Care and Use of Laboratory Animals declared by the National Institutes of Health (NIH) were followed in all the experimental procedures. Adult male Sprague–Dawley rats (weighing 120-240 g) were obtained from the Experimental Animal Center, Mansoura University, and housed under standard light conditions (12 h light/12 h dark) with food and water available ad libitum for all rats. Standard diet pellets contained not less than 20% protein, 5% fiber, 3.5% fat, 6.5% ash, and a vitamin mixture according to the standard guidelines.

### 2.4. Experimental Design

Rats were randomly classified into six groups (eight rats per group) (*n* = 8) and were injected as follows. (1) Control group: rats received distilled water (1000 mg/dl) daily for 10 days using oral gavage. (2) GL alone group: rats received *GL* (500 mg/kg/day) in distilled water (1000 mg/dl) daily for 10 days using oral gavage. (3) Cisplatin group: rats were injected with CDDP (12 mg/kg b.w, i.p) single dose on day 3. (4) CDDP+GL daily group: rats received oral *GL* (500 mg/kg/day) in distilled water daily for 10 days starting 3 days before CDDP injection (12 mg/kg b.w, i.p) single dose on day 3. (5) CDDP+GL every other day (EOD) group: rats received oral *GL* (500 mg/kg/day) in distilled water every other day for 10 days starting 3 days before CDDP injection (12 mg/kg b.w, i.p) single dose on day 3. (6) CDDP+GL inject. group: rats were injected with *GL* (500 mg/kg/day *i.p*) twice on days 2 and 6 starting 1 day before CDDP injection (12 mg/kg b.w, i.p) single dose on day 3.

Cisplatin was given as a single dose of 12 mg/kg intraperitoneally according to previous studies [41]. Also, GL doses were chosen according to previous studies [42–44]. Also, pilot experimental trials were carried out for the same purpose. Seven days post-CDDP injection (on day 10), rats were fasted for 12 hours and sacrificed by cervical dislocation. Blood serum was separated by centrifugation at 3000 rpm for 5 min and stored at −80°C for biochemical assessment. Kidney tissues were quickly harvested, and one part was instantly fixed in 10% phosphate-buffered formaldehyde for histological and immunohistochemical studies. Moreover, kidney samples were homogenized at 1 : 10 (*w* : *v*) in 0.1 M phosphate buffer (pH 7.4) with an Ultra Turrax Homogenizer and kept frozen at −80°C for antioxidant, anti-inflammatory, and apoptotic markers.

### 2.5. Histopathological Examination and Tubular Injury Score

Samples of kidney tissues of different groups were fixed in 4% paraformaldehyde for 72 hours and embedded in paraffin wax, and 3–4 *μ*m slices from the prepared paraffin blocks were stained with hematoxylin-eosin (H&E) for histopathological inspection using light microscopy according to a previous method [45]. Also, a semiquantitative scoring of the percentage of pathological injury area under a single field of vision was done and the scoring criteria was used as described previously [46, 47]: the injury area is 0%, 0 points, normal; injury area < 25%, 1 point, mild; injury area is 25–50%, 2 points, moderate; injury area is 50–75%, 3 points, severe; and injury area > 75%, 4 points, extremely severe.

### 2.6. Assessment of Renal Function Markers

Renal function markers such as BUN and serum creatinine levels were assessed colorimetrically using the commercially available kits; Biodiagnostic Assay Kit (Cairo, Egypt). Blood urea nitrogen was measured colorimetrically at 578 nm according to the previous method of Chaney and Marbach [48]. Serum creatinine was determined by measuring the colored complex formed by the reaction of creatinine with picrate in an alkaline medium colorimetrically at 520 nm according to the previous method by Schirmeister and his colleagues [49].

### 2.7. Assessment of Oxidative Stress Markers

To assess the renal oxidant status, hydrogen peroxide (H_2_O_2_) was assayed colorimetrically at 510 nm according to the method of Aebi [50]. Also, the activity of superoxide dismutase (SOD) in tissue homogenates of different groups was assessed spectrophotometrically at 412 nm using the method of DeChatelet and colleagues [51].

### 2.8. Assessment of Inflammatory and Apoptotic Markers

Renal HMGB-1 was assessed as previously described [52], using ELISA kits (Bioassay Technology Laboratory, Shanghai, China) exactly following the manufacturers' instructions. In addition, the NF-*κ*B assay kit (MyBioSource, Inc., San Diego, USA) was used to determine the renal NF-*κ*B expression. Caspase-3 was also assessed using assay kits (BioVision Inc., Milpitas, USA), according to the manufacturers' instructions.

### 2.9. Assessment of Autophagy-Related Protein, LC3 II, Using Flow Cytometry

Intracellular content of the autophagy protein, microtubule-associated protein 1A/1B-light chain 3 (LC3 II), was evaluated by flow cytometric analysis using the method of Shvets and his coworkers [53, 54]. Briefly, suspensions of cells were prepared in phosphate-buffered saline/bovine serum albumin (PBS/BSA) buffer and then incubated with anti-LC3 fluorescein isothiocyanate for 30 min at room temperature. Cells were then washed using PBA/BSA, centrifuged at 400 × g for 5 minutes, resuspended in 0.5% paraformaldehyde in PBS/BSA, and then analyzed using flow cytometry. The fluorescence-activated cell sorter (FACS) used is Becton Dickinson Accuri C6 (BD Accuri C6). The flow cytometer system is equipped with an argon-ion laser emitting at 488 nm. Data were analyzed using CellQuest software (Becton Dickinson).

### 2.10. Immunohistochemical Detection of Growth Factor, the Epidermal Growth Factor Receptor (EGFR), and Terminal Deoxynucleotidyl Transferase (TDT)

3 *μ*m thick renal sections of different groups were submerged in peroxidase for 10 minutes and washed. Then, the sections were immunostained with the primary rabbit polyclonal antibody to rat EGFR and terminal deoxynucleotidyl transferase (TDT) (Abcam, USA) at a concentration of 1 *μ*g/ml and incubated overnight at 4°C. After washing the slides with Tris-buffered saline, 100 *μ*l of poly-horseradish peroxidase (HRP) (Genemed Power-Stain 1.0 Poly HRP DAB) kits was added, incubated for 15 minutes, and rinsed 3 times with wash buffer for 2 minutes. The substrate solution was prepared by mixing diaminobenzidine (DAB) chromogen with DAB buffer solution, and then, it was added on slides and incubated for 5-10 minutes at room temperature. Slides were rinsed with tap water and counterstained with hematoxylin according to the manufacturer's instruction. Additionally, area % of immunoexpression levels of EGFR and TDT in kidney tissues was determined in 6 random fields per group using the Leica Application module attached to Full HD microscopic imaging system (Leica Microsystems GmbH, Germany).

### 2.11. Statistical Analysis

Data were expressed as the mean ± SEM. Multiple group comparisons among different groups were attained using one-way ANOVA followed by Tukey-Kramer as a post hoc test, as appropriate. *P* values < 0.05 were considered statistically significant. InStat ver. 3 software package was utilized for statistical analyses, and graphs were created by GraphPad Prism ver. 5 software (USA).

## 3. Results

### 3.1. GL Ameliorated Cisplatin-Induced Nephrotoxicity

**Figure 1:**
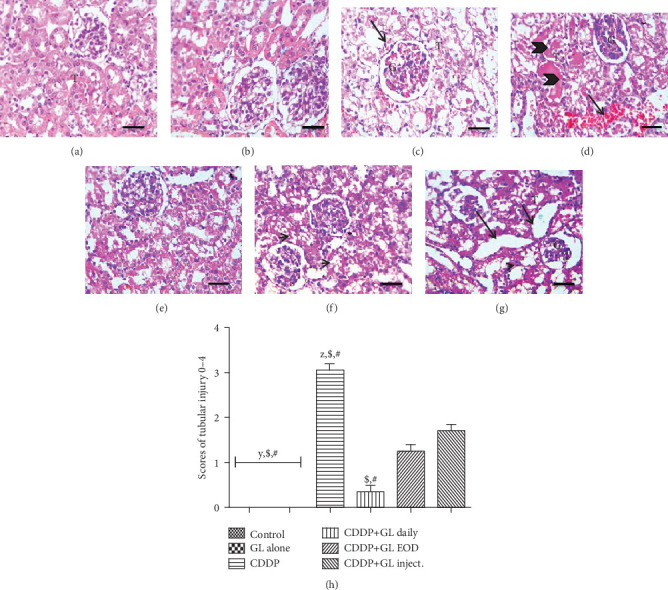
Photomicrographs of kidney sections stained with hematoxylin and eosin. (a, b) Control and G. lucidum groups show normal histology of glomeruli (G) and tubules (T). (c, d) The cisplatin group shows congested glomeruli and renal tubules (black arrow), severe tubular dilation, perivascular lymphocytic cell infiltration (G), and tubular cast formation (arrowheads). (e) The CDDP+GL daily group shows restored histological picture of the kidney. (f) The CDDP+GL EOD group shows mild vacuolar degeneration in epithelial lining renal tubules (short black arrows). (g) The CDDP+GL inject. group shows edematous swelling of Bowman's capsule of glomeruli (G), tubular dilation (long black arrows) with vacuolar degeneration in epithelial lining renal tubules (short black arrows) (T). X: 400 bar 50. (h) Statistical analysis of tubular injury scores; values are mean ± SEM (*n* = 10). ^X^*P* < 0.05 compared to the control group, ^y^*P* < 0.05 compared to the CDDP group, ^z^*P* < 0.05 compared to the CDDP+GL daily, ^$^*P* < 0.05 compared to the CDDP+GL EOD group, and ^#^*P* < 0.05 compared to the CDDP+GL inject. group using one-way ANOVA followed by Tukey-Kramer as post hoc test. CDDP: cisplatin; GL: Ganoderma lucidum; EOD: every other day.

To confirm the protective effect of GL on acute nephrotoxicity induced after CDDP, histopathological evaluation, as well as biochemical renal function indices, was carried out. Compared to the control or GL alone groups, H&E-stained kidney tissues of the CDDP group displayed massive tubular damage (showing a tubular injury score of 3 ± 0.15) including tubular dilatation, epithelial degeneration and necrosis, congestion of renal blood vessels, tubular cast formation, and perivascular lymphocytic cell infiltration (Figures 1(c), 1(d), and 1(h)). Interestingly, the tubular injury was significantly restored after GL administration daily as well as EOD showing a score of 0.35 ± 0.1 and 1.25 ± 0.16, respectively, with some mild vacuolar degeneration in epithelial lining renal tubules in the EOD group (Figures 1(e), 1(f), and 1(h)). However, in Figures 1(g) and 1(h), the photomicrograph of the GL inject. group shows edematous swelling of Bowman's capsule of glomeruli, tubular dilation with mild vacuolar degeneration in epithelial lining renal tubules, and a tubular injury score of 1.7 ± 0.21.

At the end of the experiment, on day 10, CDDP induced a distinct rise in both BUN and creatinine levels, about 5.4- and 3.8-fold as compared to the control group (Table 1). However, GL administration showed a considerable reduction in BUN levels by about 47.8% for the CDDP+GL daily group, 12% for the CDDP+GL EOD oral administration group, and only 9.8% for the CDDP+GL inject. group. On the other hand, the elevation of serum creatinine was significantly reduced after GL treatment, which was consistent with histologic improvement. The GL oral EOD and IP administration decreased serum creatinine to about 70% and 73% of the CDDP group while the CDDP+GL daily group nearly normalized serum creatinine level, 34% of the CDDP group (Table 1). Furthermore, the administration of GL alone caused no significant morphologic or biochemical alterations in renal function tests.

### 3.2. GL Reduced the Cisplatin-Induced Oxidative Stress

As shown in Table 1, kidney tissues of the CDDP group showed distinct oxidative stress that was evidenced by 0.69-fold depletion of the antioxidant enzyme, SOD, as well as a 1.8-fold increment in H_2_O_2_ renal levels as compared to the control group. Nonetheless, GL significantly counteracted the oxidative stress induced by CDDP as it upregulated the SOD levels and downregulated the H_2_O_2_ levels when compared to the CDDP group. Also, the CDDP+GL daily group showed the highest level of amelioration (reaching about 151% and 58% of the CDDP group) for SOD and H_2_O_2_, respectively. Furthermore, no significant variations have been found in the GL alone group when compared to the control group.

### 3.3. GL Decreased the Cisplatin-Mediated Inflammatory Signaling

**Figure 2:**
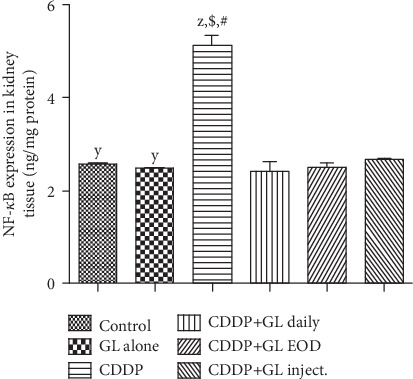
Effects of cisplatin and Ganoderma lucidum on the expression of nuclear factor-kappa B (NF-*κ*B) in kidney tissues of rats. Values are mean ± SEM (*n* = 5). ^X^*P* < 0.05 compared to the control group, ^y^*P* < 0.05 compared to the CDDP group, ^z^*P* < 0.05 compared to the CDDP+GL daily group, ^$^*P* < 0.05 compared to the CDDP+GL EOD group, and ^#^*P* < 0.05 compared to the CDDP+GL inject. group using one-way ANOVA followed by Tukey-Kramer as post hoc test. CDDP: cisplatin; GL: Ganoderma lucidum; EOD: every other day; NF-*κ*B: nuclear factor kappa B.

Inflammatory markers have been assessed using the ELISA technique for HMGB-1 and NF-*κ*B expressions in kidney homogenates of different groups. At the end of the experiment, renal HMGB-1 (Table 1), as well as NF-*κ*B (Figure 2) expressions, showed a significant upregulation in CDDP-injected rats as compared to the control group, while GL administration significantly ameliorated the CDDP-induced inflammation in terms of HMGB-1 as evidenced by 27%, 12.2%, and 10% reduction for the CDDP+GL daily, CDDP+EOD, and CDDP+GL inject. groups, respectively, being CDDP+GL daily having the lowest level of HMGB-1 when compared to the CDDP group (Table 1). Moreover, GL administration of different groups nearly normalized NF-*κ*B expressions in the kidneys when compared to the CDDP group. However, GL alone showed nonsignificant values for HMGB-1 and NF-*κ*B when compared to the control values (Table 1 and Figure 2).

### 3.4. The Apoptotic Pathway Involved in GL's Renoprotection against Cisplatin

**Figure 3:**
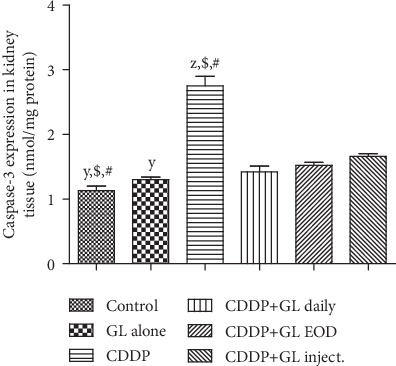
Effects of cisplatin and Ganoderma lucidum on the expression of caspase-3 in kidney tissues of rats. Values are mean ± SEM (*n* = 5). ^X^*P* < 0.05 compared to the control group, ^y^*P* < 0.05 compared to the CDDP group, ^z^*P* < 0.05 compared to the CDDP+GL daily group, ^$^*P* < 0.05 compared to the CDDP+GL EOD group, and ^#^*P* < 0.05 compared to the CDDP+GL inject. group using one-way ANOVA followed by Tukey-Kramer as post hoc test. CDDP: cisplatin; GL: Ganoderma lucidum; EOD: every other day.

**Figure 4:**
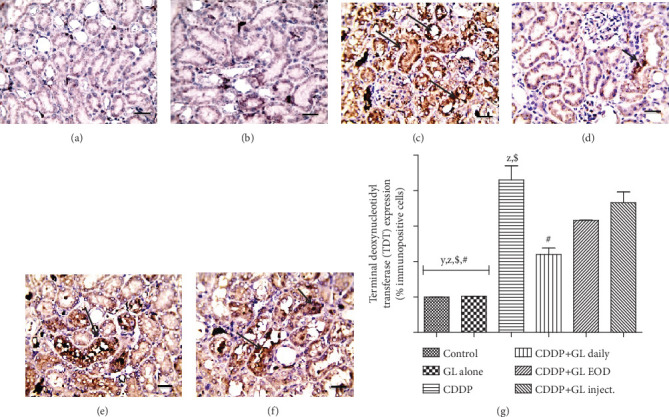
Immunohistochemical expression of terminal deoxynucleotidyl transferase (TDT). (a, b) The control and GL alone groups show very low positive stained cells. (c) The CDDP group shows very strong TDT-positive cells as indicated by intense brown color. (d) The CDDP+GL daily group shows mild TDT-positive stained cells. (e) The CDDP+GL EOD group shows moderate immunopositive stained cells as represented by positive calls. (f) The CDDP+GL inject. group shows strong TDT-positive cells with black arrows representing a high expression of TDT-positive cells. (g) Quantitative expression of TDT expressed as a percentage of immunopositive cells, values are given as mean ± SEM (*n* = 6) for each group. ^X^*P* < 0.05 compared to the control group, ^y^*P* < 0.05 compared to the CDDP group, ^z^*P* < 0.05 compared to the CDDP+GL daily group, ^$^*P* < 0.05 compared to the CDDP+GL EOD group, and ^#^*P* < 0.05 compared to the CDDP+GL inject. group using one-way ANOVA followed by Tukey Kramer as post hoc test. IHC counterstained with Mayer's hematoxylin. X: 400 bar 50. CDDP: cisplatin; GL: Ganoderma lucidum; EOD: every other day; TDT: terminal deoxynucleotidyl transferase.

One of the crucial cellular responses to kidney damage is renal cell apoptosis particularly in CDDP-induced AKI [6]. The proapoptotic caspase-3 expression levels as well as TDT have been determined to evaluate the antiapoptotic effect of GL treatment in CDDP-injured rats (Figures 3 and 4). Regarding caspase-3 expression, an intense upregulation in CDDP-injected rat kidneys, 2.4-fold of the control group, was detected (Figure 3). After GL administration, caspase-3 expression levels significantly decreased (reaching about 0.5-, 0.55-, and 0.66-fold of the CDDP group) in the daily, EOD, and inject. groups, respectively. Moreover, the results from TDT assay confirmed that CDDP could induce extensive renal cell apoptosis (Figure 4(c)); nevertheless, treatment with GL greatly reduced the number of TDT-positive apoptotic cells which confirmed that GL mitigated the CDDP-induced tubular cell apoptosis as shown in Figures 4(d)–4(f).

### 3.5. GL Inhibits Autophagy-Related Protein in Cisplatin-Induced Nephrotoxicity

**Figure 5:**
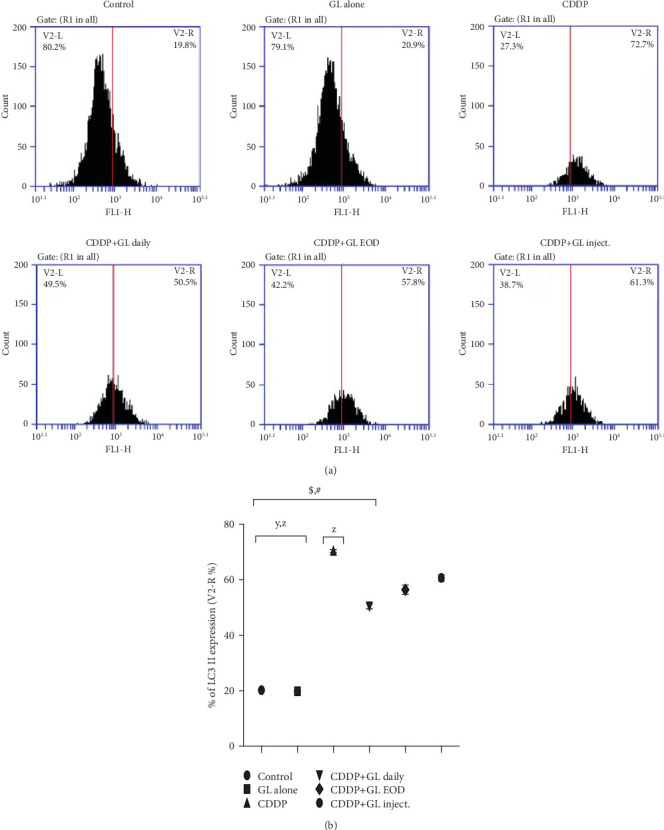
Effect of Ganoderma lucidum and cisplatin on the expression of LC3 II in kidney tissue by flow cytometry. (a) Photos from flow cytometry show different treatment groups of LC3 II expression in renal tissues. (b) Quantitative expression of LC3 II in different treatment groups as a percentage (V2-R%); ^X^*P* < 0.05 compared to the control group, ^y^*P* < 0.05 compared to the CDDP group, ^z^*P* < 0.05 compared to the CDDP+GL daily group, ^$^*P* < 0.05 compared to the CDDP+GL EOD group, and ^#^*P* < 0.05 compared to the CDDP+GL inject. group using one-way ANOVA followed by Tukey-Kramer as post hoc test. CDDP: cisplatin; GL: Ganoderma lucidum; EOD: every other day; LC3 II: microtubule-associated protein 1A/1B-light chain 3.

To elucidate whether GL played any role in autophagy-mediated nephrotoxicity in rats, the autophagy-related protein was analyzed using flow cytometry. As displayed in Figure 5, the accumulation of LC3 II was detected after CDDP treatment 352% as a percentage of the control group. This increment in autophagy-related protein was decreased by the administration of GL daily and EOD by 28% and 20%, respectively. Moreover, IP injection with GL did not significantly decrease the LC3 II protein when compared to the CDDP group (Figure 5).

### 3.6. GL Downregulates the Epidermal Growth Factor (EGFR) Expression in Cisplatin-Treated Kidneys

**Figure 6:**
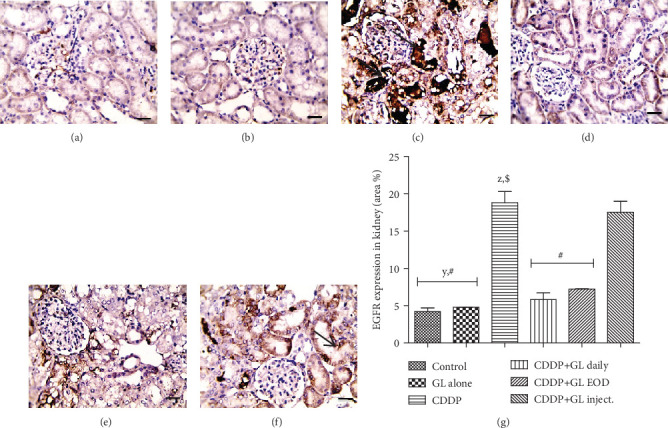
Expression of epidermal growth factor receptor (EGFR) by immunohistochemical staining. (a, b) The control and GL alone groups display a minimal expression of EGFR. (c) The cisplatin group displays an immense EGFR expression (brown staining). (d) The CDDP+GL daily group displays a limited EGFR expression. (e) The CDDP+GL EOD group displays a minimal EGFR expression. (f) The CDDP+GL inject. group displays EGFR expression. (g) Area percentage of immunopositive reaction. Values are given as mean ± SEM (*n* = 6) for each group. ^X^*P* < 0.05 compared to the control group, ^y^*P* < 0.05 compared to the CDDP group, ^z^*P* < 0.05 compared to the CDDP+GL daily group, ^$^*P* < 0.05 compared to the CDDP+GL EOD group, and ^#^*P* < 0.05 compared to the CDDP+GL inject. group using one-way ANOVA followed by Tukey-Kramer as post hoc test. IHC counterstained with Mayer's hematoxylin. X: 400 bar 5. CDDP: cisplatin; GL: Ganoderma lucidum; EOD: every other day; EGFR: epidermal growth factor receptor.

Cisplatin injection caused marked 4.4-fold upregulation in EGFR renal expression as compared to that of the control group (Figures 6(c) and 6(g)). Administration of GL daily and every other day downregulated the renal expression of EGFR to 0.3- and 0.38-fold of the CDDP group, respectively, as shown in Figures 6(d), 6(e), and 6(g). However, GL inject. rats did not show any significant difference in renal EGFR expression when compared to the CDDP group (Figures 6(f) and 6(g)).

## 4. Discussion

Cisplatin is a highly effective antineoplastic drug, which is widely used in the treatment of several types of cancers, the second leading cause of global death. However, CDDP causes acute nephrotoxicity. Despite the use of adjuvant therapies, such as those with fluids and mannitol, nephrotoxicity remains a significant limiting factor, and novel adjuvant treatments need to be developed [16]. This study suggested the potential nephroprotective effect of GL against CDDP-induced acute nephrotoxicity in rats. Moreover, our study has a novelty in investigating the possible underlying mechanisms including its effect on the oxidative and inflammatory status as well as apoptosis. Moreover, the possible roles of autophagy and epidermal growth factor receptor downstream signaling pathways in CDDP-induced renal injury were also explored.

To induce acute nephrotoxicity in rats, CDDP was injected intraperitoneally as a single dose of 12 mg/kg. Acute nephrotoxic damages were confirmed by increased levels of nephrotoxicity indices including BUN and serum creatinine as well as the histopathological changes. BUN and serum creatinine are known to accumulate in the blood when the kidneys fail to clear nitrogenous wastes as a result of extensive morphological damage and functional impairment [55]. Our findings confirmed those of previous studies [56–58]. Notably, the administration of GL had hampered the renal damages produced by CDDP, as evidenced by decreased BUN and serum creatinine levels. Also, histopathological examination showed complete restoration of the severe tubular dilation and epithelial degeneration induced by CDDP in the daily GL-treated group which was superior over the other two groups.

Besides nephrotoxicity indices, the possible mechanisms underlying the nephroprotective effects of GL were investigated. There are undeniable shreds of evidence that oxidative stress plays a pivotal role in the pathogenesis of CDDP-induced nephrotoxicity. It has been reported that the generation of reactive oxygen species (ROS) induced by CDDP promotes cellular damage [59, 60], apoptosis, and inflammation [61]. In this study, the CDDP-injected group showed serious oxidative stress in renal tissues indicated by SOD reduction and a marked increase in H_2_O_2_ as well as lipid peroxidation. These findings were in accordance with those demonstrated by previous studies [62, 63]. Pretreatment with GL normalized the levels of the oxidative markers in renal tissues compared to the CDDP group. Indeed, dietary antioxidants had been used to ameliorate the CDDP-induced renal injury in rats [64, 65]. In addition to the oxidative stress, the role of inflammation in CDDP-induced nephrotoxicity is well documented. Cisplatin-induced cellular damage and necrosis lead to the release of damage-associated molecular pattern molecules, such as HMGB-1 [13], which bind to toll-like receptor (TLR) and induce inflammation through activation of the NF-*κ*B pathway [66, 67]. Our results showed that GL downregulated the HMGB-1/NF-*κ*B signaling pathway augmented by CDDP. Thus, we suggested that GL may have ameliorated the CDDP-induced AKI through inhibition of the HMGB-1/NF-*κ*B-mediated inflammatory pathway.

Moreover, apoptosis and autophagy play crucial roles in AKI development; however, the exact pathogenesis of AKI induced by CDDP remains poorly understood. Counteracting effects of autophagy on cell survival and death have been documented, and it has been suggested that autophagy could promote cell survival only when there is an appropriate level of autophagy, while it mediates cell death when autophagy flux is very high [68]. Accordingly, we assessed the changes in LC3 II protein levels, a molecular marker of autophagy [69], and results showed marked promotion of LC3 II expressions in the CDDP group but were inhibited in GL-exposed rats. The CDDP+GL daily group showed superior activity over both the EOD and inject. groups. However, it is still significantly higher than the control rats. Moreover, apoptosis is a programmed cell death that could be elicited by oxidative stress, inflammation, and many other factors, and it is controlled by the balance between the pro- and antiapoptotic genes [70]. Treatment with GL greatly reduced the expression of caspase-3 and the number of TDT-positive renal cells which indicated that GL could ameliorate the CDDP-induced AKI through effective prevention of apoptosis in CDDP-exposed renal tissues as previously mentioned in cyclophosphamide-induced AKI [71]. Moreover, to further elucidate the proved complex interaction between autophagy and apoptosis in CDDP-induced nephrotoxicity [72], we noticed that the expressions of key apoptotic caspases, caspase-3, as well as TDT, were inhibited and accompanied by downregulation in autophagy-related protein, LC3 II, indicating that the occurrence of apoptosis may be regulated by autophagy. Furthermore, we suggested that CDDP caused excessive ROS production (high H_2_O_2_ levels) where antioxidants such as SOD would be consumed destroying the balance between ROS and antioxidant systems leading to autophagy-mediated apoptosis. Kang et al. [71] have documented that the combined action of oxidative stress imbalance and autophagy defects causes abnormal cell apoptosis which leads to the occurrence of AKI [71].

Indeed, it is undeniable that the disruption of growth factors and their receptors play a role in CDDP-induced renal injuries [56]. In renal proximal tubular cells, CDDP also activated the EGFR, which is involved in cell death rather than survival under these conditions [73]. EGFR may mediate renal injury by induction of inflammatory factors and cell apoptosis. However, inhibition of EGFR showed therapeutic potential for AKI during endotoxemia and diabetic nephropathy [74, 75]. Our results showed that the upregulation of EGFR, following CDDP injection, was significantly downregulated in rats treated with GL, and this correction was associated with the improvement that occurred in nephrotoxicity indices. Our results were in accordance with a previous study which showed that erlotinib would protect against CDDP-induced AKI through inhibition of the EGFR-Akt signaling pathway [34]. Therefore, we suggested that GL protection against CDDP-induced nephrotoxicity might be through inhibition of the downstream signaling of EGFR. Furthermore, some studies reported that the EGFR blockade may also indirectly protect against diabetic nephropathy by increasing islet prosurvival autophagy activity [74]. In this current study, we found that EGFR signaling blockade by GL was accompanied by a significant reduction in the pro-cell death persistent autophagy and apoptosis induced by CDDP; however, this autophagy activity was still higher than that in the control group. This means that GL may have inhibited the EGFR signaling, which in turn would inhibit the high autophagy flux augmented by CDDP; however, GL remained the appropriate prosurvival autophagy activity to guard against CDDP-induced cell death. Further investigations are clearly warranted to deeply elaborate the interrelationship between the inhibition of autophagy-mediated apoptosis and EGFR signaling. Finally, no studies have reported a decrease in CDDP chemotherapeutic effect in patients that concomitantly received GL extract; however, a very recent study has reported that GL extracts may sensitize cancer cells to conventional chemotherapeutics [76]. The present study supports further those clinical studies on GL and suggested some molecular mechanisms whereby GL would protect from CDDP-induced nephrotoxicity.

## 5. Conclusion

Taken together, the results of the current study suggested that GL might have a renoprotective effect in acute nephrotoxicity induced by CDDP. This effect might be attributed to the antioxidant, anti-inflammatory effect, and attenuation of autophagy-mediated apoptosis in renal tissues, and it has coincided with inhibition of the HMGB-1/NF-*κ*B and EGFR signaling pathways. Additionally, we concluded that daily administration of GL was superior in the postulated renoprotective effects over both the EOD group and the inject. group. Furthermore, GL could be considered as a renoprotective natural compound in CDDP-induced kidney injury, although further studies are required to confirm its beneficial effects in patients. However, it has been proved that GL does not affect the anticancer potential of CDDP.

## Figures and Tables

**Table 1:** Effects of cisplatin and Ganoderma lucidum on nephrotoxicity indices, oxidative stress, and inflammatory markers in kidney tissues of rats.

	BUN (mg/dl)	Serum creatinine (mg/dl)	SOD (ng/mg protein)	H_2_O_2_ (ng/mg protein)	HMGB-1 (pg/mg protein)
Control	13.10 ± 1.30^y,z,$,#^	0.32 ± 0.02^y,$,#^	144.20 ± 2.40^y^	0.15 ± 0.01^y^	4.40 ± 0.11^y,z,$,#^
GL alone	12.12 ± 0.65^y,z,$,#^	0.31 ± 0.02^y,$,#^	145.68 ± 0.38^y^	0.16 ± 0.01^y^	5.06 ± 0.09^y,$,#^
CDDP	71.24 ± 1.52^z,$,#^	1.2 ± 0.10^z,$,#^	100.90 ± 9.21^z,$,#^	0.29 ± 0.02^z,$,#^	7.40 ± 0.14^z,$^
CDDP+GL daily	37.79 ± 1.87^$,#^	0.41 ± 0.02^$,#^	151.07 ± 1.91	0.17 ± 0.01	5.42 ± 0.14^$,#^
CDDP+GL EOD	62.17 ± 1.52	0.84 ± 0.06	138.75 ± 5.93	0.19 ± 0.01	6.51 ± 0.24
CDDP+GL inject.	64.55 ± 1.24	0.88 ± 0.09	129.28 ± 7.63	0.20 ± 0.01	6.64 ± 0.17

Values are mean ± SEM (*n* = 5). ^X^*P* < 0.05 compared to the control group, ^y^*P* < 0.05 compared to the CDDP group, ^z^*P* < 0.05 compared to the CDDP+GL daily group, ^$^*P* < 0.05 compared to the CDDP+GL EOD group, and ^#^*P* < 0.05 compared to the CDDP+GL inject. group using one-way ANOVA followed by Tukey-Kramer as post hoc test. CDDP: cisplatin; GL: Ganoderma lucidum; EOD: every other day; BUN: blood urea nitrogen; SOD: superoxide dismutase; H_2_O_2_; hydrogen peroxide; HMGB-1: high-mobility group box-1.

## Data Availability

All data generated or analyzed during this study are included in this published article.

## Abbreviations

CDDP: Cisplatin
GL: Ganoderma lucidum
SOD: Superoxide dismutase
ROS: Reactive oxygen species
EGFR: Epidermal growth factor receptor
HMGB-1: High-mobility group box-1
NF-*κ*B: Nuclear factor kappa B
LC3 II: Microtubule-associated protein 1A/1B-light chain 3
TDT: Terminal deoxynucleotidyl transferase.
